# Wind Turbines: A Different Breed of Noise?

**DOI:** 10.1289/ehp.122-A20

**Published:** 2014-01-01

**Authors:** Nate Seltenrich

**Affiliations:** Nate Seltenrich covers science and the environment from Oakland, CA. His work has appeared in *High Country News*, *Sierra*, *Earth Island Journal*, the *San Francisco Chronicle*, and other local and national publications.

Sue Hobart and her husband built their dream home in 2007 on a quiet, wooded lot outside Falmouth, Massachusetts. Five years later they abandoned it. Less than 1,500 feet from the empty house stands a mammoth wind turbine erected three years ago by Notus Clean Energy. Three blades mounted upon the 262-foot tower sweep an area of the sky equal to 1.3 acres, the size of a football field. They are visible through the forest from the house’s meticulously landscaped yard.

But the problem with the property wasn’t the degraded view—at least not for the Hobarts. The problem was the noise. Shortly after the turbine switched on in 2010, Sue began experiencing headaches, dizziness, insomnia, and a ringing in her ears. When she noticed the symptoms briefly disappeared during trips out of town, she began attributing them to the arrival of the turbine. Within two years she was ready to leave.

Fellow Falmouth resident Annie Hart Cool can relate. “We live on two and a half acres of land, and we can’t use it because of the noise,” she says. Cool and her husband live near one of two city-owned turbines installed in 2010 and 2011 that power a nearby wastewater treatment facility, with the excess energy providing a source of revenue for the city. “We were all so excited about it until it turned on, and then we realized we couldn’t live with it,” Cool says.

In all, 41 Falmouth families have formally complained to city leaders—as have countless other wind-farm neighbors in countries including Australia, Canada, and England. And a small but growing body of evidence suggests that the health impacts of wind farms can be very real.

## Environmental Noise and Health

Researchers have been studying the impacts of environmental noise on human health since at least 1930.^1^ Varying degrees of evidence exist for a wide range of nonauditory health effects potentially stemming from noise exposures, including cardiovascular disease,^2^^,^^3^^,^^4^ hypertension,^5^^,^^6^ stroke,^7^^,^^8^ diabetes,^9^ sleep disturbance,^10^ endocrine effects,^11^^,^^12^ minor psychiatric disorders,^13^ and impaired cognitive development.^14^

Yet a March 2013 report by ENNAH, the European Network on Noise and Health, identified 12 areas in which the science of nonauditory health effects of noise still lacks sufficient evidence.^15^ These include the extent to which air pollution and other coexposures may contribute to health effects identified in urban noise studies, the comparative health effects of short- and long-term noise exposures, and the relationship between individual health outcomes and noise sensitivity. “Noise sensitivity” has been defined multiple ways but generally refers to an individual’s increased likelihood of perceiving noises as annoying—i.e., the person is both more attuned to and more bothered by noise.^16^

Although investigators may not know the exact nature of the relationship between noise and health impacts, or why noise affects some people differently than others, the evidence to date suggests that environmental noise pollution can have serious implications for public health. After air pollution, traffic noise is the second-largest environmental factor affecting human health in the European Union and Norway, according to a 2011 report by the World Health Organization.^17^

The report authors estimate that each year, western Europeans lose 1.0–1.6 million disability-adjusted life-years (DALYs) due to traffic noise, a figure thought to be conservative despite accounting for impacts on cardiovascular disease, cognitive impairment in children, sleep disturbance, tinnitus, and annoyance. Sleep disturbance was determined to be responsible for the largest independent share of DALYs lost (903,000), and annoyance (654,000) the next-largest share.^17^

Based on its standing definition of health as “a state of complete physical, mental, and social well-being and not merely the absence of disease or infirmity,” the WHO concludes that noise-induced annoyance “may be considered an adverse effect on health.”^17^ High levels of annoyance have also been shown to lead to stress responses and sleep loss, including attendant symptoms such as headache, gastrointestinal upset, anxiety, fatigue, and hypertension.^18^^,^^19^^,^^20^

Much of what scientists can conclude today about the health effects of noise in general draws upon studies of transportation noise in urban areas conducted over the past four decades. Among the first to suggest a link between noise and learning impairment was a 1975 study by environmental psychologist Arline Bronzaft.^21^ In a New York City elementary school adjacent to an elevated train track, Bronzaft compared the reading scores of children in classrooms facing the tracks to those of children in classrooms on the other side of the building. She discovered that children on the noisy side were nearly one year behind their peers in reading. After two years, once noise-abatement measures had been completed—and other classroom variables held constant—Bronzaft returned to the school and found reading scores on both sides of the building to be at the same grade level.^22^

Today, notwithstanding Bronzaft’s groundbreaking early study and New York City’s ongoing efforts to mitigate noise pollution, much of the field’s cutting-edge research originates outside the United States, where there is more funding and interest surrounding the nonauditory health effects of environmental noise. For instance, a landmark study dubbed HYENA (Hypertension and Exposure to Noise near Airports) assessed the relationship between noise from aircraft and road traffic near airports and its implications for hypertension. Over a period of four years, researchers measured blood pressure and collected a range of health, socioeconomic, and lifestyle metrics via questionnaire from 4,861 individuals between the ages of 45 and 70. These participants had lived near one of six major European airports for at least five years. The study revealed clear relationships between risk of hypertension and both nighttime aircraft activity and average daily road noise, after adjusting for major confounders including age, sex, body mass index, alcohol intake, and physical activity.^23^

## Wind Turbines

Large-scale wind turbines are a relatively recent innovation, so the body of peer-reviewed research addressing the potential impacts of their unique brand of sound is sparse and particularly unsettled. Anecdotal evidence strongly suggests a connection between turbines and a constellation of symptoms including nausea, vertigo, blurred vision, unsteady movement, and difficulty reading, remembering, and thinking.^24^

The polarizing issue of wind-turbine noise is often framed one of two ways: Turbines are either harmless,^25^ or they tend to have powerful adverse effects, especially for sensitive individuals.^26^ According to Jim Cummings, executive director of the nonprofit Acoustic Ecology Institute in Santa Fe, New Mexico, most of the reports to date that have concluded turbines are harmless examined “direct” effects of sound on people and tended to discount “indirect” effects moderated by annoyance, sleep disruption, and associated stress. But research that considered indirect pathways has yielded evidence strongly suggesting the potential for harm.

Multiple recent studies, including one coauthored by Daniel Shepherd, senior lecturer at New Zealand’s Auckland University of Technology, have demonstrated that sleep interference gets worse the nearer residents are to turbines.^20^^,^^27^ “Sleep is absolutely vital for an organism,” he says. “When we lose a night’s sleep, we become dysfunctional. The brain is an important organ, and if noise is disturbing its functioning, then that is a direct health effect.”

In another recent study, Shepherd made a case for approaching the debate from a social or humanistic standpoint, taking perceived effects seriously even if the potential mechanisms through which they occur remain unclear.Many reasons exist for taking this approach with wind-turbine noise, he wrote.^28^

First is that turbine noise (that is, the aerodynamic noise produced by air moving around the spinning blades as opposed to any mechanical noise from the motor itself) is often deemed more annoying than the hum or roar of transportation noise because of its repetitive nature and high variability in both level and quality—from “swoosh” to “thump” to silence, all modulated by wind speed and direction. This pulsing, uneven quality enables the noise to repeatedly capture the attention and become more difficult to ignore.^29^^,^^30^

In addition, unlike vehicle traffic, which tends to get quieter after dark, turbines can sound louder overnight. As Cummings explains, “Often at night, wind shear sets in. This creates conditions with moderate winds at hub height and a sharp boundary layer below which winds are much lower, or even near still.” The absolute noise level of the wind farm may be no more than during the day, but it can be 10–20 decibels louder than the quieter nighttime ambient sound levels. This detail has important implications for sleep disruption.

Third, wind turbines generate lower frequencies of sound than traffic. These lower frequencies tend to be judged as more annoying than higher frequencies and are more likely to travel through walls and windows.^31^ Infrasound, or sound frequency lower than 20 Hz—inaudible to the human ear—has been associated in some studies with symptoms including fatigue, sleeplessness, and irritability,^32^ as well as with changes to the physiology of the inner ear that have poorly understood implications.^33^ Many previous infrasound studies have looked at exposures in populations such as jet pilots and factory workers. Today, Cummings says, “There are some studies looking at whether wind turbine infrasound may have specific qualities that make it more apt to trigger health effects, especially nausea, than ‘normal’ infrasound from wind or waves or traffic, but these are still very preliminary.”

Likely exacerbating the issue, Shepherd notes, residents of rural and semirural areas like Falmouth may be a self-selected group who are naturally more sensitive to noise than the population at large. As such, they may have greater expectations of quiet and be more aware of noise disturbances, amplifying the potential for health effects related to environmental noise.^34^

“People live in these areas and create their own little patches of paradise, and part of that is the soundscape,” Shepherd says. “When an industrial noise source comes in, they get very stressed, because they’re losing something that is very dear to them.” The negative feelings engendered by this loss of “amenity” (something that once brought joy) can further contribute to a feedback loop of stress, sleep loss, negative emotions, and related health impacts.^10^^,^^35^

But are quiet-seeking rural dwellers more prone to report health impacts from new turbines simply because they anticipate a negative outcome? That’s the question surrounding the role of the “nocebo” effect—the flip side of placebo, where negative thoughts engender negative outcomes—which is yet another point of contention in the turbine-noise debate. The turbine nocebo effect gained currency worldwide following the March 2013 release of two Australian reports claiming to offer evidence that people who expect adverse effects of turbines—in part as a result of activism by groups such as Australia’s Waubra Foundation—are more likely to report having them.

In Cummings’ estimation, the two new studies are not as definitive as they purport to be.^36^ One, a paper published at the University of Sydney,^37^ considered no explanation of health effects other than nocebo. The other, a peer-reviewed study published in *Health Psychology*,^38^ reported expectations to have, at most, a very small effect on either the number or severity of reported symptoms.^36^ Still, the nocebo effect, whose role has been established in other areas of epidemiology and medicine,^39^ may be impossible to rule out as at least a partial factor in some neighbor responses.

## Looking Long Term

The gold standard for proving causality of an exposure is the randomized clinical trial. But when it comes to testing the health effects of noise exposure on humans, such a study design is likely to be not only impractical and difficult to implement, but also unethical.

The next-best evidence would come from longitudinal field research, many researchers agree, such as long-term studies that assess the health of a community before a turbine project is ever proposed and then continue to follow up during operation. Lercher notes that some effects of chronic noise exposure such as elevated blood pressure could take one or two decades to manifest at significant levels.

Most of the studies performed to date around both transportation and wind-farm sources have been cross-sectional, which makes it impossible to assess causality. That’s because investigators cannot establish whether the potential cause precedes the potential effect. Lercher stresses that cross-sectional studies purporting to demonstrate a relationship between noise exposures and health effects may be averaging out potential effects that are only visible in some subgroups—e.g., those with certain medical risk factors, or those exposed to the noise for longer than others.

Today, wind turbine noise is attracting ever more interest as a public health issue. That’s evident in the offerings at Noise-Con, an annual conference dedicated to noise research, says Purdue University professor Patricia Davies. She chaired the 2013 conference, which was organized in conjunction with the International Wind Turbine Noise Conference in Denver, Colorado. Davis says Noise-Con is beginning to see nearly as many sessions organized around wind turbine noise as in all categories of transportation noise combined. “A few years ago, there were just occasional papers,” she says. “Certainly there’s more interest right now, because of course there have been a lot more wind turbines built.”

Despite increased attention to the issue throughout Falmouth, some residents claim they’re hardly better off today than they were when the first turbine switched on in March 2010. Once complaints about the turbines reached a fever pitch, the city voted to limit operation to 16 hours a day,^40^ and in November 2013 a judge reduced operations further, to 12 hours a day, Monday through Saturday.^41^ The two city-owned turbines still follow that schedule after surviving a recent petition to decommission them, and in spite of not generating enough income to cover operating costs. Their future remains uncertain.
